# The tau hypothesis of nodding syndrome in Africa

**DOI:** 10.1371/journal.pntd.0011526

**Published:** 2023-08-17

**Authors:** Michael S. Pollanen, Sylvester Onzivua

**Affiliations:** 1 Centre for Research in Neurodegenerative Diseases and Department of Laboratory Medicine and Pathobiology, University of Toronto, Ontario, Canada; 2 Department of Pathology, Mulago Referral Hospital, Kampala, Uganda; IRCCS Sacro Cuore Don Calabria Hospital, ITALY

## Introduction

Nodding syndrome (NS) was first described in the Mahenge mountains in Tanzania, where it exists in an endemic form of epilepsy [1]. In her pioneering work in Tanzania, Louise Jilak-Aall described NS as a pediatric seizure disorder and later observed parkinsonian-like manifestations in some adult survivors with NS [1]. NS later emerged in an epidemic form in both South Sudan and northern Uganda. In South Sudan, Spencer and colleagues [2] reported an association of NS with onchocerciasis but later attributed this to a coincidental opportunistic infection. In Uganda, the NS epidemic coincided with regional insurgency by the Lord’s Resistance Army and displacement of families into refugee camps with the occurrence of new cases of NS peaking in the mid-2000s [3].

NS usually begins with spells of head bobbing [4–7], which have now been determined to represent atonic seizures. Clinical presentation with nodding is typically at 5 to 15 years of age with the progression to grand mal seizures within months or years of the initial presentation. Many children with NS have a clinical course dominated by grand mal seizures. However, some children also develop marked neurocognitive and motor decline. Some individuals with NS develop marked impairment of speech with mutism, and other can develop frontal lobe disinhibition (e.g., coprophagia) [8]. It is unknown if NS is invariably fatal, but when death occurs, it is often related to accidental drowning during seizures, status epilepticus, or neurologic decline with malnutrition and pressure ulcers [8].

The brain in NS shows varying degrees of brain atrophy, cerebellar degeneration, white matter degeneration, and microglial activation (neuroinflammation) [9]. The brain also has intraneuronal deposition of an abnormally phosphorylated protein: the microtubular associated protein tau [8]. In fatal cases of NS, tau pathology is variably abundant in the brain with preferential involvement of the frontal neocortex.

### Potential multifactorial pathogenesis

Epidemiological studies on NS in Uganda have disclosed correlation between parasitic infection [10], malnutrition [11], environment (insurgency and civilian displacement) [3], and young age. These associations suggest potential multifactorial pathogenesis for NS. The exclusive occurrence of NS in the young likely relates to a selective vulnerability present at a certain stage of neurodevelopment in early childhood, but the precise details are unclear. Furthermore, NS often affects more than one sibling in a family, but not the parents. This suggests that common environmental factor(s) could be responsible for NS, rather than a mendelian disorder.

A causal link between NS and onchocerciasis has been proposed [9]. This is supported by two lines of observations. First, there is a partial overlap in the geographical distribution of onchocerciasis and NS in Africa. Second, many individuals with NS are seropositive for ov16, indicating exposure to *Onchocera volvulus* (OV). On the basis of current research, two main hypotheses have emerged to explain NS. These two hypotheses essentially rest on different interpretations about the interplay between OV, tau, and seizures.

### Two hypotheses: OV and tau

**Fig 1:**
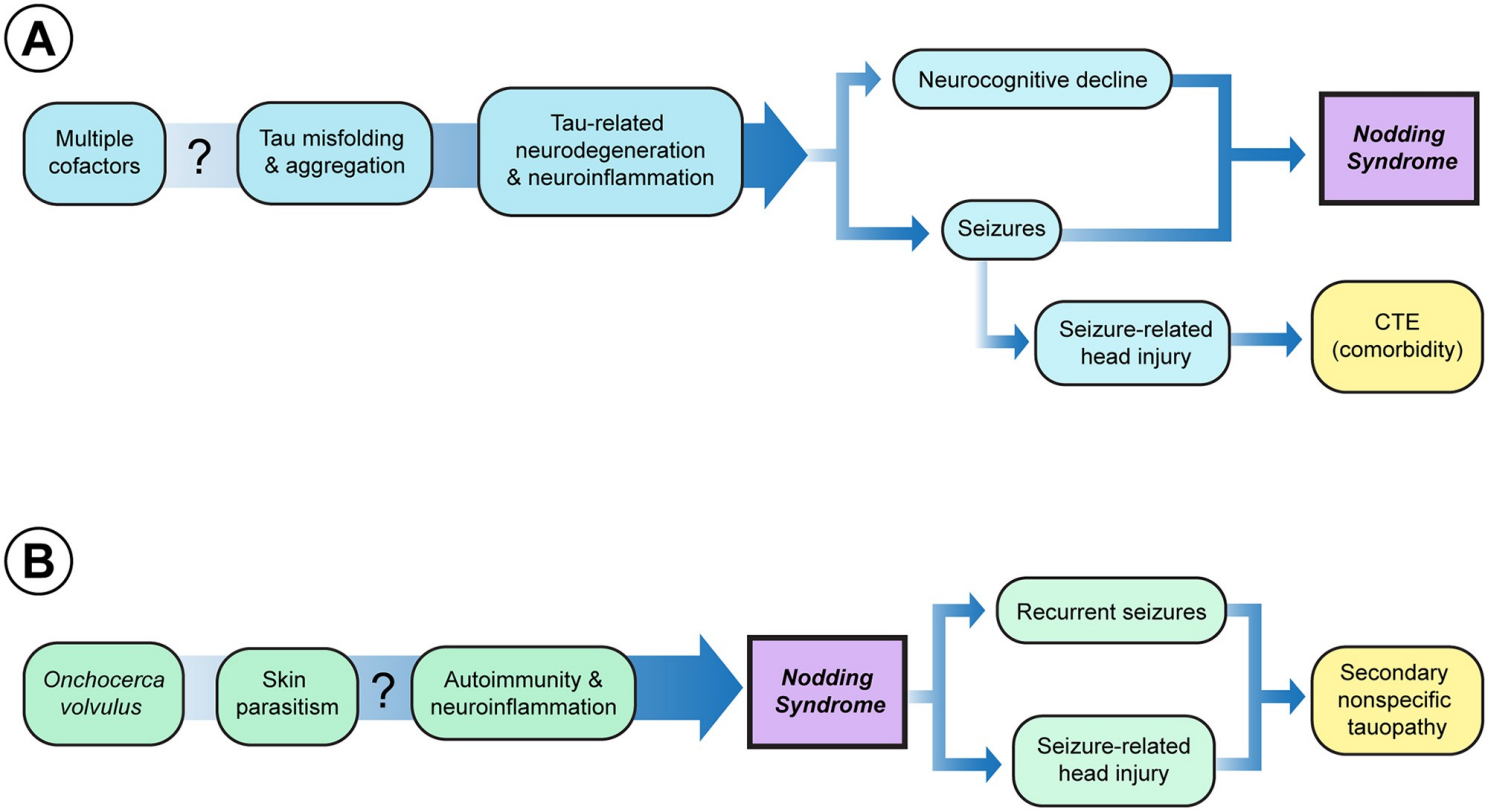
Tau and OV hypotheses for nodding syndrome. **(A) Tau hypothesis.** Risk factor results in tau misfolding and aggregation in the brain. The tau cascade causes neurodegeneration and neuroinflammation, leading to the seizures and neurocognitive decline in NS. Seizure-related head injury could also cause comorbid CTE, possibly contributing to disability. **(B) OV hypothesis.** Extracerebral OV infection leads indirectly to autoimmunity/neuroinflammation that causes seizures by an unknown mechanism. The repeated seizures cause brain insults. The recurrent seizures and seizure-related head injuries both lead to nonspecific tau pathology. CTE, chronic traumatic encephalopathy; NS, nodding syndrome; OV, *Onchocera volvulus*.

The two main hypotheses for NS are the tau hypothesis and the OV hypothesis (Fig 1). The tau hypothesis is that the cause of NS is unknown, but the pathophysiology of the disease process results in tau pathology, which then causes both the seizures and the neurological decline observed in many cases of NS (Fig 1A). The OV hypothesis suggests that OV somehow causes seizures, and the seizures secondarily result in tau deposition in the brain (Fig 1B).

Fig 1 compares the OV and tau hypotheses. There are three major differences between the OV and tau hypotheses. First, the OV and tau hypotheses are based on differing mechanisms of pathogenesis. Second, the tau hypothesis is open to a combination of risk factors causing NS and does not postulate an initial cause of the disease. Thus, the tau hypothesis makes no statement about the cause of NS; the tau hypothesis only makes a claim about pathogenesis. Specifically, the claim is that tau is involved in the pathogenesis of the disease as an intermediate step. Third, the key difference is that the OV hypothesis suggests that tau is a seizure-related epiphenomenon, whereas the tau hypothesis suggests a key role for tau in pathogenesis.

At this time, there is sufficient published data to compare the OV and tau hypotheses. This can be achieved by identifying the strengths and gaps in each hypothesis. This analysis is described below.

#### OV hypothesis

It has been suggested that OV nematode could cause NS by direct or indirect mechanism(s). The direct mechanism requires a neuroinvasive nematode infection. The indirect mechanism could involve molecular mimicry-associated autoimmunity related to primary OV infection. Since it is well known that OV is a cutaneous pathogen, it is implausible to propose that neuroinvasion of OV causes NS. Furthermore, OV has not been demonstrated in NS brains. Indeed, this is sufficient evidence to exclude a neuroinvasive nematode infection because nematode infections of the brain are readily apparent on neuropathologic examination, even remotely after the primary infection. In addition, the proposal of Johnson and colleagues [12] that leiomodin-1 antibodies causes an OV-mediated autoimmune epilepsy syndrome in NS have not been substantiated by Hotterbeekx and colleagues [13]. Despite this, autoimmunity (related to OV or another pathogen) as a pathogenic mechanism for NS has not been entirely excluded. But, if autoimmunity does play a role in NS, then a plausible pathogen and a verifiable autoantigen must be demonstrated. In addition, neuroinflammation could play a role in NS, but it is unclear how to link neuroinflammation with OV, in the absence of neuroinvasive infection.

A priori, the epidemiological association of OV and NS can either be causal or coincidental. The companion *Viewpoint* provides the details of the onchocerciasis hypothesis.

#### Tau hypothesis

It is widely accepted that tau misfolding and aggregation is a fundamental driver in many aging-related neurodegenerative diseases [14]. On this basis, the presence of tau pathology could explain disease progression in NS. The tau hypothesis proposes that the presence of tau pathology in NS is an obligate characteristic of the disease. The tau hypothesis suggests that tau-related neurodegeneration could be an explanation for the clinical features of NS. Specifically, tau pathology could explain the clinical features of NS, including seizures (cortical epileptogenic foci). Interestingly, the presence of tau deposition in the substantia nigra in some Ugandan NS cases correlates with the observation of parkinsonian-like features in long-term Tanzanian NS survivors. This observation also predicts that some of the long-term survivors in the aging Ugandan NS cohort will develop parkinsonism.

The main criticism of the tau hypothesis is that tau deposition perhaps could be secondarily caused by repeated seizures. This is theoretically supported by the presence of tau deposition in the temporal lobe in some cases of temporal lobe epilepsy. However, the distribution and nature of tau pathology in the NS brain is entirely different from that reported in temporal lobe epilepsy [15], thus excluding any significant similarity. In addition, the neuropathology of idiopathic epilepsy is well characterized and has been studied for decades and does not correlate with tau pathology.

It has also been suggested that subclinical cumulative head injury (concussion) secondary to repeated seizures could result in spurious tau pathology in NS. The tau pathology related to repeated head injury is known as chronic traumatic encephalopathy (CTE) [16]. The neuropathology of NS is substantially dissimilar from CTE. The neuropathologic hallmark of CTE is the perivascular accumulation of tau at the depths of sulci. The distribution of tau pathology in NS is mostly in the superficial layers of the neocortex at the crowns of gyri. Therefore, the distribution of tau pathology in NS is literally the anatomical opposite of CTE. On this basis, the current state of the available evidence does not support that the fundamental tau pathology is due to CTE. Despite this, a definitive role for tau pathology as the main driver of disease in NS has not been conclusively proven. The role of recurrent seizure-related head injury causing CTE as a comorbidity, and contributing to clinical decline, must also be further explored.

On balance, while it is true that tau pathology is present in NS, the mechanism of deposition and precisely how it relates to disease progression are yet to be determined. For example, it will be important to determine if tau pathology in NS progresses in stages over the duration of illness, as it does in Alzheimer’s disease. The main gap in the tau hypothesis is that tau pathology is not etiologically specific. Therefore, the presence of tau pathology cannot be used to directly infer the cause of NS. More specifically, the presence of tau pathology in NS does not exclude OV as a cofactor in NS. On a logical basis, the OV hypothesis and the tau hypothesis are not mutually exclusive. For example, if OV is a causal factor that can indirectly induce tau aggregation via an extracerebral mechanism (e.g., autoimmunity), then the tau cascade could, in theory, be initiated by OV. The main difference in this unified OV-tau hypothesis is that tau still plays a fundamental role in pathogenesis, rather than simply representing an epiphenomenon. Furthermore, another pathogen, not necessarily OV, could also initiate the tau cascade.

### Future research

Future research will need to address the disparities between the OV and tau hypotheses. This requires definitive proof or refutation that OV causes NS (e.g., actually demonstrate how a skin parasite causes a brain disease). Otherwise, the potentially coincidental association of OV with NS justifies a cautious approach to the OV hypothesis. The proponents of the tau hypothesis are also required to show how tau aggregation causes brain dysfunction in NS. An open mind and balanced approach that embraces both the OV and tau hypotheses are required to seek the truth about this enigmatic disease.

**Key Learning Points**

- Nodding syndrome (NS) is a neglected tropical neurological disease in Africa.
- Abnormal intraneuronal aggregates of the microtubule-associated protein tau are found in the NS brain, but there is also an association between NS and onchocerciasis.
- Two main hypotheses have emerged to explain NS: the tau hypothesis and the *Onchocerca volvulus* (OV) hypothesis.
- The tau hypothesis is that the cause of NS is unknown but that the pathophysiology of the disease results in tau pathology, which explains the seizures and other clinical hallmarks of NS.
- The OV hypothesis suggests that OV causes NS but that tau deposition in the brain is incidental.
- The gap in the OV hypothesis is that it is mechanistically unclear how a skin parasite can cause a brain disease. In contrast, tau pathology is known to cause neurodegeneration, but further studies must elucidate what causes tau misfolding in NS.
